# Getting a Whiff of Speciation by Reinforcement

**DOI:** 10.1371/journal.pbio.0020454

**Published:** 2004-11-23

Creating a new species is a bit like climbing a greased flagpole—it's hard to get started and even harder to keep going. Random genetic variations may introduce slight differences between two groups but, without some means to keep them apart, sexual interbreeding will quickly remix the genes and obliterate the differences. Accidents of geography—the rising of mountains or a course-changing river, for instance—can provide physical isolation, which then enables genetic divergence through the accumulation of mutations, either through natural selection or genetic drift.

In contrast, speciation without geographic separation relies on the direct action of natural selection to complete the speciation process by strengthening behavioral differences, a process called reinforcement. One of the most powerful means of completing speciation is through the evolution of mate discrimination.

A study by Daniel Ortiz-Barrientos and colleagues focuses on the genetic underpinnings of mate discrimination in Drosophila. They identify two loci that influence the likelihood that a female will choose to mate with a conspecific male, rather than one of a closely related species.

Drosophila pseudoobscura and D. persimilis exist together along the west coast of the United States (sympatry), but separately elsewhere (allopatry). When together, they hybridize and produce sterile males. While D. pseudoobscura males will court females of both species, females prefer conspecific males. This female preference is stronger in sympatric females, an enhancement that presumably evolved by the direct action of natural selection to prevent females from wasting their reproductive efforts producing sterile sons. This variation allowed the authors to conduct a series of genetic crosses among flies of the same species but from different locations. Because the daughters of discriminating D. pseudoobscura females were just as discriminating as their mothers, Ortiz-Barrientos and colleagues concluded that female mating discrimination was inherited as a dominant trait. Further crosses showed that genes responsible for female preference were on the X and fourth chromosomes, and high-resolution mapping refined their locations sufficiently to allow the identification of likely candidate genes. While more work remains to be done, the most promising genes in both regions appear to be involved with olfaction. The fact that one of them, *CG13982*, is known to up-regulate the other, *bru-3*, strengthens the case that these are indeed promising candidates.[Fig pbio-0020454-g001]

**Figure pbio-0020454-g001:**
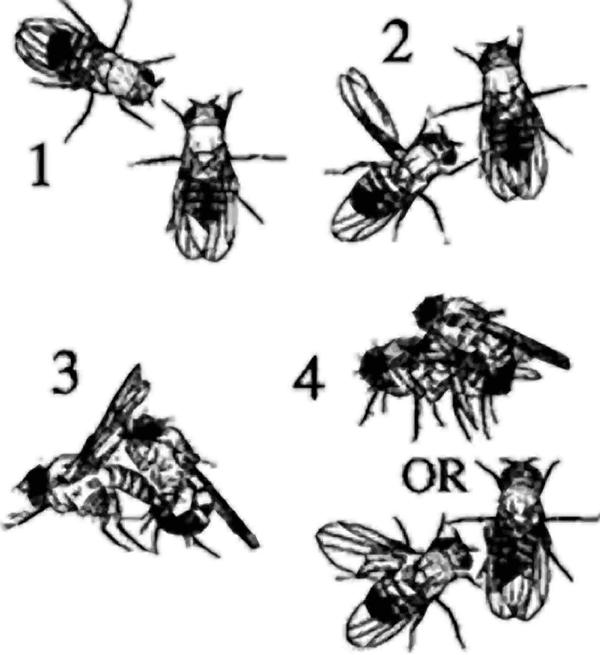
Drosophila choosing their mates

According to their findings, the authors propose a novel model of mating discrimination in D. pseudoobscura based on the combined response to auditory and olfactory cues. The first of these two layers, weak, or “basal” mating discrimination, has previously been associated with a set of traits for acoustic recognition and mapped to chromosomal regions that are inverted between the two species. Such inversions prevent recombination from purging alleles, thereby contributing to hybrid male sterility. As a consequence, when these species interbreed, they inexorably produce sterile males. The second layer, elucidated in the current study, is “reinforced” mating discrimination, which appears to be related to olfactory cues. This additional system of discrimination helps the first layer to fully eliminate the inevitable cost of producing sterile males. Once the nascent species have started up that slippery pole, reinforced discrimination could provide the traction needed to reach its top.

